# Quality evaluation of extracted ion chromatograms and chromatographic peaks in liquid chromatography/mass spectrometry-based metabolomics data

**DOI:** 10.1186/1471-2105-15-S11-S5

**Published:** 2014-10-21

**Authors:** Wenchao Zhang, Patrick X Zhao

**Affiliations:** 1 Plant Biology Division, The Samuel Roberts Noble Foundation, 2510 Sam Noble Parkway, Ardmore, OK 73401, USA

**Keywords:** Metabolomics, Liquid chromatography/mass spectrometry, Extracted ion chromatogram (EIC), Chromatographic peak, Quality evaluation

## Abstract

**Background:**

Extracted ion chromatogram (EIC) extraction and chromatographic peak detection are two important processing procedures in liquid chromatography/mass spectrometry (LC/MS)-based metabolomics data analysis. Most commonly, the LC/MS technique employs electrospray ionization as the ionization method. The EICs from LC/MS data are often noisy and contain high background signals. Furthermore, the chromatographic peak quality varies with respect to its location in the chromatogram and most peaks have zigzag shapes. Therefore, there is a critical need to develop effective metrics for quality evaluation of EICs and chromatographic peaks in LC/MS based metabolomics data analysis.

**Results:**

We investigated a comprehensive set of potential quality evaluation metrics for extracted EICs and detected chromatographic peaks. Specifically, for EIC quality evaluation, we analyzed the mass chromatographic quality index (MCQ index) and propose a novel quality evaluation metric, the EIC-related global zigzag index, which is based on an EIC's first order derivatives. For chromatographic peak quality evaluation, we analyzed and compared six metrics: sharpness, Gaussian similarity, signal-to-noise ratio, peak significance level, triangle peak area similarity ratio and the local peak-related local zigzag index.

**Conclusions:**

Although the MCQ index is suited for selecting and aligning analyte components, it cannot fairly evaluate EICs with high background signals or those containing only a single peak. Our proposed EIC related global zigzag index is robust enough to evaluate EIC qualities in both scenarios. Of the six peak quality evaluation metrics, the sharpness, peak significance level, and zigzag index outperform the others due to the zigzag nature of LC/MS chromatographic peaks. Furthermore, using several peak quality metrics in combination is more efficient than individual metrics in peak quality evaluation.

## Background

One of the critical tools for effective metabolomics studies is liquid chromatography/mass spectrometry (LC/MS). LC/MS is a sensitive technique that separates chemical mixtures based on their physical properties and evaluates their mass to identify the species present. To perform the mass spectrometry, the sample must first be ionized. LC/MS utilizes electrospray ionization (ESI) rather than electron ionization (EI), used in gas chromatography/mass spectrometry (GC/MS). The 'spray' technique produces relatively high quality mass spectra, but often fails to generate distinct peaks on the total ion current (TIC) traces. Efficient methods of extracting the selected or extracted ion chromatograms (EICs) and distinguishing the analyte peaks by inspecting the chromatograms at appropriate m/z values need to be developed [1-4]. Currently, EICs can be extracted by binning the data points in two-dimensional space (m/z and scan number) into each centroid mass with a specific tolerance [5] or by tracing the mass slices with several continuous scans using advanced pattern recognition or video processing-inspired object tracing approaches [6,7]. The binning method is a simple, direct method; however, it suffers when an m/z larger than the fixed tolerance drifts between scans and can split a single analyte signal into two neighboring bins. On the other hand, the tracing method can resolve the splitting issue; however, it may produce low-quality extracted EICs displaying high noise and background levels that weaken or bury meaningful analyte peaks. This is due to contaminants along with other factors such as the LC mobile phase, atmospheric environment, or solvent types [8-10]. In the worst cases, the extracted EICs contain nothing but background and noise. Therefore, efficient methods to evaluate extracted EIC quality and filter out the "bad" EICs before the downstream time-consuming peak detection processing are highly desired.

The extracted EIC can contain multiple peaks with similar m/z values, but different retention times, possibly due to the presence of isomers. Detecting peaks, especially exactly finding the analyte related chromatographic peaks and acutely locating their elution starts and ends, from the EICs is another critical step in LC/MS-based metabolomics data analysis. Chromatographic peaks can be detected by directly analyzing the local maximum points [11], matching chromatographic peaks with the second derivative of the Gaussian function using a fixed window width [5,6], or analyzing the EIC's two dimensional continuous wavelet transform (2D CWT) coefficients [6,12]. The local maximum point detection-based method frequently overestimates the number of detected peaks and the matched Gaussian filter approach only can detect peaks with fixed widths. Although the 2D CWT methods are promising, LC/MS chromatographic peaks still present a challenge due to limited scan number (usually 5-20 scans) and the common zigzag peak shape. This makes low signal-to-noise ratio (SNR) peaks even more difficult to be detected. Additionally, when the spectra are transformed from continuous profile mode into centroid mode, spikes with only one or several continuous scans are common and difficult to be distinguished from authentic analytical peaks [13]. Therefore, efficient methods to evaluate the detected chromatographic peak's quality and filter out the "bad" chromatographic peaks prior to downstream processing are also highly desired.

In this paper, we investigated potential metrics for evaluating extracted EIC and detected chromatographic peak quality. Specifically, for extracted EICs, we analyzed the mass chromatographic quality index (MCQ index) [8] and proposed a novel EIC quality evaluation metric, named the EIC-related global zigzag index, based on the EIC's first order derivatives. We also analyzed and compared a comprehensive set of chromatographic peak quality metrics including sharpness, Gaussian similarity, SNR, peak significance level, triangle peak area similarity ratio (TPASR), and local peak-related zigzag index. We conducted both case studies and comprehensive performance evaluations of these metrics. The case study-based evaluations were conducted by analyzing several representative EICs and chromatographic peaks with challenging features common to metabolomics data. The comprehensive metric evaluations were performed on a complete data set. During data processing, the metric cutoff thresholds were varied, both individually and in combination, followed by calculating the Recall, Precision, and F-Score for the whole dataset. The case study-based evaluation was used to evaluate the metric's performance against specific, known issues, whereas the comprehensive evaluation was used to show the overall performance.

Based on the case-specific and comprehensive evaluations and analyses of the extracted EICs, we concluded that the MCQ index is more suitable for selection and alignment of analyte components, but cannot fairly evaluate EICs with high background signals or with only a single peak. Our proposed EIC related zigzag index can efficiently evaluate both scenarios. In the case- specific and comprehensive evaluations and analyses of the chromatographic peaks, the sharpness, peak significance level, and zigzag index outperformed the other three metrics due to the zigzag nature of LC/MS peaks. Furthermore, combining several peak quality metrics proved to be more efficient than using a single metric for chromatographic peak quality evaluation.

## Methods

The extracted EIC can be represented by its specific m/z value; however, it is possible to have multiple peaks due to isomers or individual analyte components with common fragments, which need a further peak detection procedure. The detected chromatographic peaks can be derived from biologically meaningful analytes, and also can be from chemical noise, which are usually represented by its specific m/z, the position of its apex, and the left and right boundaries.

Therefore, in LC/MS metabolomics data analysis, development of effective quality evaluation metrics for both EICs and chromatographic peaks is necessary and highly desired. In the following subsections, we will provide detailed descriptions of the metrics used to evaluate the extracted EICs and detected chromatographic peaks.

### Quality evaluation metrics for extracted EICs

**MCQ index**. LC/MS uses ESI techniques, which commonly result in high background levels and spike noise in the chromatograms. The spikes can be detected by calculating the similarity index between the original and its smoothed version, which is sometimes referred to as the spike detection index. The background can be detected by the calculating the similarity index between the original and its mean-subtracted version, which is sometimes referred to as the background detection index. The MCQ index[8] incorporates the two similarity indexes by calculating the similarity between the original mass chromatogram and both the smoothed and mean-subtracted versions. Currently, the MCQ index is widely used for noise reduction and candidate component detection in LC/MS data analysis, particularly for chromatographic alignment [10,14].

**Global zigzag index**. The extracted EICs and their local chromatographic peaks commonly display a zigzag shape. Here we propose a new metric, named the "zigzag index", to measure the degree of EIC zigzag. Compared to local chromatographic peaks, the EIC zigzag metric is a global index used to evaluate the degree of zigzag in the extracted EICs. Suppose the extracted EIC intensities are represented by *N *data points as *I*_1_, *I*_2_, ... , *I_n-1_ , I_n_ , I_n+1_*, ... , *I_N _*the procedure to calculate the zigzag index is as follows:

1) Calculate the effective peak intensity by subtracting the baseline at the peak apex:

(1) $$EPI=Max\left(I_{1},I_{2},\ldots ,I_{n-1},I_{n},I_{n+1},\ldots ,I_{N}\right)-Baseline\left(Apex\right)$$

2) Calculate the EIC's first-order derivative and acquire the increment for each data point pair:

(2) $$d_{n}=I_{n}-I_{n-1},d_{n+1}=I_{n+1}-I_{n};n=2,3\ldots N$$

3) Calculate the variance between each neighbor increment pair:

(3) $$v\left(d_{n},d_{n+1}\right)={\left(d_{n}-d_{nmean}\right)}^{2}+{\left(d_{n+1}-d_{nmean}\right)}^{2}\;\text{and}\;d_{n_{mean}}=\frac{\left(d_{n}+d_{n+1}\right)}{2.0}$$

4) Using formula (2), the variance can be represented as:

(4) $$v\left(d_{n},d_{n+1}\right)=0.5*{\left(2I_{n}-I_{n-1}-I_{n+1}\right)}^{2}$$

Where (2*I_n _*- *I_n-1 _*- *I_n+1_*)^2 ^indicates the local zigzag degree of data point *I_n-1_*,*I_n_* and *I_n+1_*, then the zigzag sum is:

(5) $$Sum\text{\_}zig\text{\_}zag=\sum_{n=2}^{n=N-1}{\left(2I_{n}-I_{n-1}-I_{n+1}\right)}^{2}$$

5) Calculate the average and normalized zigzag index:

(6) $$Zigzag\text{\_}index=\frac{\sum_{n=2}^{n=N-1}{\left(2I_{n}-I_{n-1}-I_{n+1}\right)}^{2}}{N*EPI^{2}}$$

From a mathematical viewpoint, the MCQ index is defined by the statistical distribution and can approximate global quality, whereas the zigzag index is defined according to the continuous transition of neighboring points, measuring shape quality. Additionally, the MCQ index has a dynamic range between 0 and 1.0, while the zigzag index has a dynamic range between 0 and 4.0.

Based on the indexes above, an extracted EIC quality filtering procedure can be determined. An extracted EIC can be considered high quality only if the calculated MCQ index is higher than a user-specified threshold or if the calculated global zigzag index is lower than a user-specified threshold.

### Quality evaluation metrics for detected chromatographic peaks

**Sharpness**. Suppose the detected peak profile intensities between its left and right boundaries are represented as *I*_1_, *I*_2_, . . . , *I_p-1_ , I_p_ , I_p+1_*, . . . , *I_N_*. Where *N *is the total data point number and *p *is the peak apex index. The sharpness of the detected chromatographic peak is defined as follows [15,16].

(7) $$Sharpness=\sum_{i=2}^{p}\frac{I_{i}-I_{i-1}}{I_{i-1}}+\sum_{i=p}^{N-1}\frac{I_{i}-I_{i+1}}{I_{i+1}}$$

**Gaussian similarity**. The ideal chromatographic peak can be estimated by classical or modified Gaussian functions [17]. The Gaussian similarity is calculated from the detected peak intensities' dot product and Gaussian curve fitting. This is used to evaluate the symmetric quality of the detected chromatographic peaks [16].

**SNR**. Signal-to-noise ratio (SNR) is a relative criterion, usually defined in the wavelet domain and estimated based on the high and low frequency peak signal components [18,19]. Usually, SNR is estimated by the ratio of the continuous wavelet transform (CWT) coefficient at a marker point to the 95% quantile of the absolute CWT coefficient at scale 1 [18].

**Peak significance level**. Peak significance level[20] is defined by the ratio between the mean intensity of data points near the peak apex and the mean intensity of data points near the two boundaries.

**TPASR**. The Triangle Peak Area Similarity Ratio(TPASR)[20] is defined as follows:

(8) $$\left\{\begin{array}{c} TPA=0.5*Peak\text{\_}Width*Intensity\left(Peak\text{\_}Apex\right)\\ RPA=\overset{Right\text{\_}Boundary}{\underset{i=Left\text{\_}Boundary}{\sum }}Intensity\left(i\right)\\ TPASR=\frac{\left|TPA-RPA\right|}{TPA} \end{array}\right)$$

TPASR provides an index for the proximity of the detected real peak and the triangle peak connected by the apex and two boundaries. A TPASR value close to 0 indicates a better peak quality.

**Local zigzag index**. Similar to the global zigzag index that can evaluate the zigzag degree of the extracted EIC, a local zigzag index can be used to evaluate the zigzag degree of local detected chromatographic peak. The calculation procedure is identical to the EIC global zigzag index and lower zigzag index values denote higher peak quality.

Of the six metrics, Gaussian similarity, SNR, peak significance level, and TPASR can be used to evaluate the chromatographic peak quality from a macro viewpoint, whereas the sharpness and local zigzag index evaluate quality from a micro viewpoint. Combined, these criteria provide a more comprehensive evaluation of detected chromatographic peak quality.

## Results and analysis

**Figure 1:**
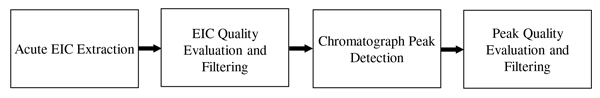
**Flowchart of the data processing program**. The program consists of four modules: acute EIC extraction, EIC quality evaluation and filtering, chromatographic peak detection, and peak quality evaluation and filtering.

In order to evaluate the efficiency of the metrics for the extracted EIC and the detected chromatographic peak, all of the EIC's and peak's chromatogram data points should be provided. However, the analytical output from existing LC/MS data analysis tools including XCMS [5], MZmine [11], and MAVEN [7] only provide peak feature's information. Therefore, a data processing platform that is compatible with the existing tools and capable of extracting and accessing chromatographic data points was required. To solve this issue, we developed our own data processing program in Matlab, which consists of four sequential modules: 1) acute EIC extraction, 2) EIC quality evaluation and filtering, 3) chromatographic peak detection, and 4) peak quality evaluation and filtering (Figure 1).

Of the four modules, the mass trace method was adopted for acute EIC extraction. This is based on region of interest (ROI) detection in a two-dimensional scan or in retention time vs. m/z space. A CWT-based method was adopted for chromatographic peak detection. The mathematical principles for these methods are similar to those adopted in the latest XCMS.

**Figure 2:**
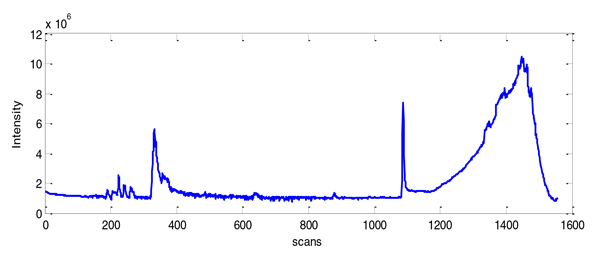
**TIC of the analyzed UPLC/MS profile**.

To evaluate the effectiveness of our software and metrics, a real LC/MS profile dataset was generated using an Agilent HPLC system interfaced with a quadrupole-time-of-flight (Q-TOF) Premier mass spectrometer. Figure 2 shows the total ion chromatogram (TIC) of the analyzed ultra-performance liquid chromatography (UPLC)/MS profile.

Using our developed data processing program and the LC/MS profile dataset, case-specific and comprehensive analysis and evaluation for the extracted EIC and the detected chromatographic peak quality metrics were implemented. In the following subsections, we will describe our case-specific and comprehensive evaluation results.

### Case-specific evaluation and analysis

### Case evaluations and analysis by representative EICs

**Figure 3:**
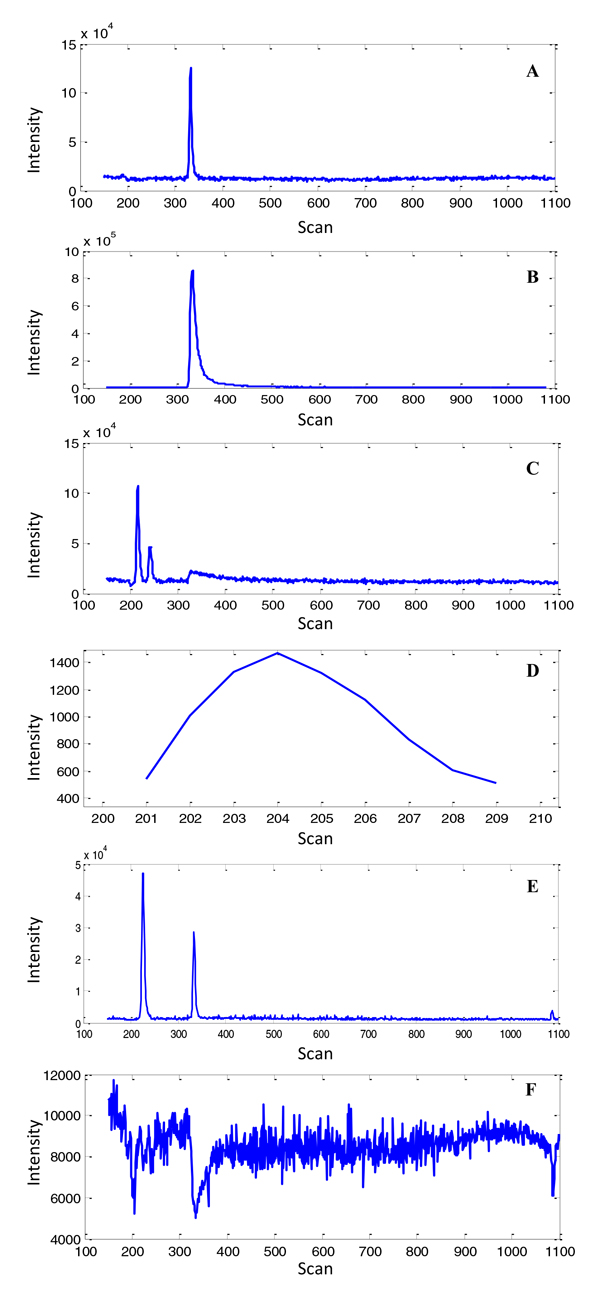
**Plots of six representative EICs**. The extracted EICs are represented with their specific m/z and unique EIC_ID: (A) [72.0810, 27], (B) [79.1092, 40], (C) [90.0552, 90], (D) [110.168,134], (E) [118.188,154], and (F) [121.158 167].

In our Matlab-based data processing program, the main parameter for the mass shift tolerance was configured to 40 ppm. In total, 611 EICs were extracted. The MCQ index and global zigzag index were calculated to evaluate the EIC quality. Figure 3 shows six representative extracted EICs and their metric measurements are given in Table 1.

**Table 1:** Metric measurements of six representative extracted EICs.

Extracted EIC with		Spike Detection Index	Background Detection Index	MCQ Index	Zigzag Index
				
*EIC ID*	*Central m/z*				
27	72.0810	0.9952	0.4895	0.4798	0.0034
40	79.1092	0.9976	0.9742	0.9717	0.0003
90	90.0552	0.9934	0.4637	0.4563	0.0057
134	110.168	0.9910	0.3360	0.3115	0.0289
154	118.188	0.9879	0.8942	0.8807	0.0004
167	121.158	0.9984	0.1024	0.0855	0.5562

Figure 3 andTable 1 demonstrate several types of observations commonly found in the LC/MS dataset. The EIC with EIC_ID = 40 (Figure 3B) is an obviously good EIC. It has good chromatographic shape with low background and no obvious spike noise. Its evaluation metrics produced a relatively high MCQ index and low zigzag index indicating that it is a high quality EIC and can be used as a reference chromatographic peak for alignment. The EIC with EIC_ID = 167 (Figure 3F) is an obviously "bad" EIC and accordingly its evaluation metrics produced a relatively low MCQ index and high zigzag index. The EICs with EIC_ID = 27 (Figure 3A) and EIC_ID = 90 (Figure 3C) contain relative high background signals, possibly due to solvent contaminants. This resulted in a relatively low background detection index and MCQ index. The EICs with EIC_ID = 90 (Figure 3C) and EIC_ID = 154 (Figure 3E) have more data points and contain multiple peaks, whereas the EIC with EIC_ID = 134 (Figure 3D) only contains few data points of one single peak. Despite their high background or containing few data points, they can still be considered "good" EICs due to their fair local chromatographic peak shape. However, the evaluation metrics produced relatively low background detection indices and MCQ indices, indicating that these EICs are "bad." Conversely, the zigzag index for these EICs was relatively low, indicating high quality EICs. In fact, only the obviously "bad" EIC with EIC_ID = 167 produced a relatively high zigzag index. Therefore, we concluded that the MCQ index cannot fairly evaluate EIC quality, especially in cases with high background or containg only single peak. However, our proposed zigzag index was able to fairly evaluate those EICs.

### Case evaluations and analysis by representative chromatographic peaks

**Figure 4:**
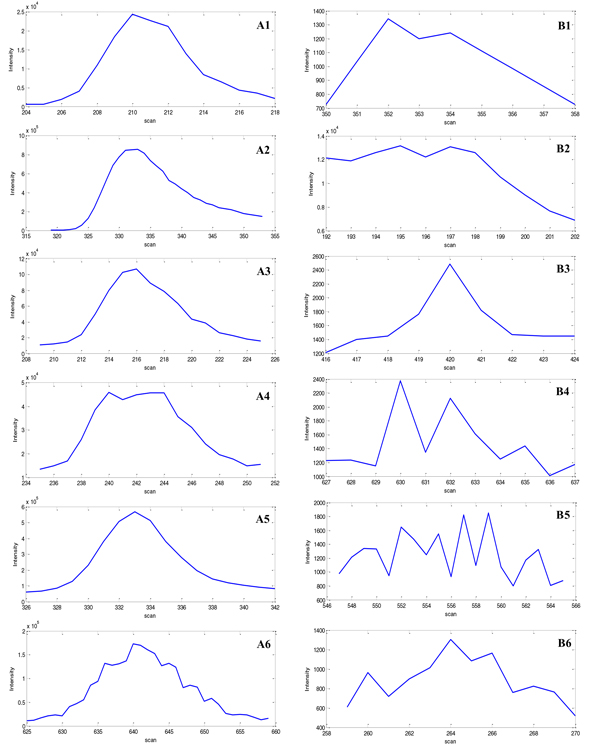
**Plots of six representative "good" and "bad" detected chromatographic peaks**. The "good" and "bad" chromatographic peaks are labeled as group 'A' and 'B', and also represented with the specific EIC_ID and apex elution's scan number: (A1) [36,210], (A2) [40,334], (A3) [90,216], (A4) [90,243], (A5) [153,333], and (A6) [205,640]. (B1) [61,352], (B2) [90,197], (B3) [154,420], (B4) [154,630], (B5) [211,559], and (B6) [338,264].

A CWT-based peak detection method was adopted to detect meaningful chromatographic peaks from the extracted EICs. Frequently LC/MS produces some "good" chromatographic peaks that should be considered for downstream analysis and some noisy or low quality peaks that should be avoided. In this study, we aimed to identify a metric or metric combination that could distinguish between "good" and "bad" peaks. After consulting with experienced experts, twelve peaks were selected for our case-specific evaluations, consisting of six representative "good"' and "bad" peaks each (Figure 4).

The six representative "good" peaks included ideal peaks with nice characteristics such as being smooth and symmetrical and those having lower background. It also included some non- ideal peaks including those with high background/baseline, asymmetrical peak shape with a long tail, a sudden intensity drop near the apex position, or a generally good profile but with a zigzagging shape. Six representative "bad" peaks with equivalent characteristics were also selected. These representative "good" and "bad" peaks were evaluated using the previously described peak quality metrics. Table 2 and Table 3 show the evaluation metrics for the "good" and "bad" peaks, respectively.

**Table 2:** Metric measurements of six representative "good" chromatographic peaks

Detected Chromatographic Peaks				Sharpness	Gaussian Similarity	SNR	Peak Significance	TPASR	Zigzag Index
						
*EIC ID*	*Apex m/z*	*Left boundary*	*Right boundary*						
36	76.0394	204	218	8.9705	0.9166	8.0778	12.7402	0.1566	0.0176
40	79.1103	319	353	11.3840	0.6215	25.843	10.9250	0.1286	0.0011
90	90.0550	209	225	5.1435	0.9113	7.1372	7.0022	0.1905	0.0110
90	90.0554	235	251	2.6920	0.9450	7.5643	3.9047	0.0783	0.0245
153	118.086	326	342	5.0117	0.8970	9.4289	7.1237	0.2745	0.0082
205	133.106	625	659	6.4712	0.9674	23.1665	12.6363	0.1683	0.0166

**Table 3:** Metric measurements of six representative "bad" chromatographic peaks.

Detected Chromatographic Peaks				Sharpness	Gaussian Similarity	SNR	Peak Significance	TPASR	Zigzag index
						
*EIC ID*	*Apex m/z*	*Left boundary*	*Right boundary*						
61	81.2037	350	358	1.3857	0.9204	1.7314	1.4284	0.2824	0.1146
40	90.0552	192	202	0.7868	0.9708	3.0460	1.3116	0.4495	0.0849
154	118.194	416	424	1.4336	0.7430	1.8057	1.4668	0.0134	0.2167
154	118.195	627	637	2.1550	0.6153	2.0857	1.3998	0.2241	0.7840
211	134.987	547	565	2.8075	0.5157	2.2730	1.3840	0.5540	0.9587
338	190.253	259	270	2.1482	0.8328	6.6839	1.5912	0.1919	0.3399

**Figure 5:**
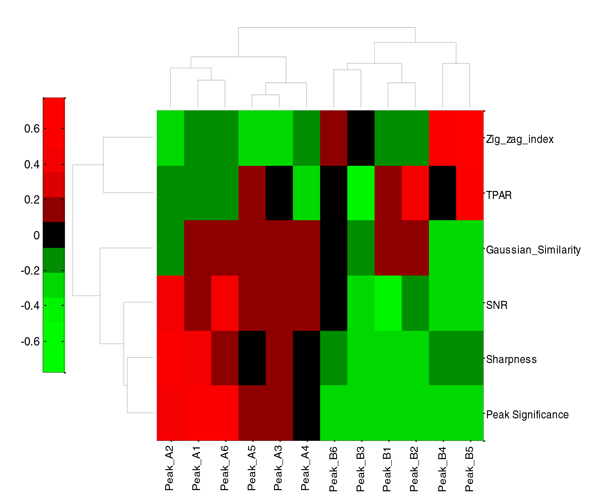
**Clustering analysis results for the "good" and "bad" representative chromatographic peaks**. The good peaks are labeled Peak_A1-Peak_A6 and the "bad" peaks are labeled Peak_B1- Peak_B6. The clustering analysis was performed using all of the six peak quality evaluation metrics: peak significance, sharpness, SNR, Gaussian similarity, TPASR, and zigzag index.

To further validate the peak quality evaluation metrics, all of the metric values for the "good" and "bad" peaks were subtracted by their mean values and normalized to [-1.0 ~ 1.0] by dividing their maximum value, respectively. We then performed a clustering analysis on the twelve peaks using all six normalized metric values (Figure 5**)**.

As can be seen in Figure 4, there were some obvious differences between the "good" and "bad" representative peak groups, but there were also some less significant differences between the "good" peaks. However, looking at the evaluation metrics in Tables 2 and 3, it was not easy to distinguish "good" from "bad" peaks based on one or two individual metrics. For example, peak_A2 (EIC_ID = 40, apex_scan = 334) had lower Gaussian similarity due to asymmetric peak shape and peak_A4 (EIC_ID = 90, apex_scan = 243) had relatively low sharpness and peak significance due to the intensity drop at the peak apex. It was difficult to judge the quality of peak_B6 (EIC_ID = 338, apex_scan = 264) from SNR because it was comparable to other good peaks; however, the peak had excessive zigzagging and a relative low signal intensity, detected by the zigzag index and peak significance level, which appropriately designated it low quality peak.

The evaluation metric clustering analysis showed that the six "good" and six "bad" representative peaks were clearly clustered into two groups (Figure 5**)**. In addition, we observed some information redundancy and correlations between the peak significance, sharpness and SNR metrics. An increased peak significance also showed increased sharpness and SNR. Taken together, these results suggested that good chromatographic peaks should have a relatively high sharpness, Gaussian similarity, SNR, and peak significance level, but a relatively low TPASR and zigzag index. Using one or two individual metrics would be insufficient to fully evaluate chromatographic peak quality. However, combining the proposed metrics can efficiently distinguish "good" peaks from "bad" peaks.

### Comprehensive evaluation and analysis

The existing tools for LC/MS-based metabolomics data analysis generally aim to identify biological meaningful peaks while filtering out as many noisy peaks as possible. The processing modules in the currently available tools include EIC quality filtering and chromatographic peak filtering. This is usually achieved by simply comparing them based on some threshold or criteria; therefore, the evaluation methods and cutoff thresholds greatly affect the final peak detection performance.

Currently, there is no comprehensive evaluation of final peak detection performance with the adopted evaluation metrics and their cutoff thresholds. Additionally, authentic chromatographic peaks may come from mixtures of metabolites or solvents, producing divergent observed peaks from identical data sets between different tools. To conduct a comprehensive evaluation and analysis of the metrics proposed in this study related to final peak detection performance, we needed an efficient method to define the "ground truth peaks." Here, we adopted the strategy proposed by Tautenhahn et al. [6] where a peak is considered a ground truth peak if it can be detected by multiple tools. Three open-source tools, XCMS [5], MZmine [11], and MAVEN [7], were employed. These tools are widely used in LC/MS metabolomics data analysis and always can provide reliable analysis results.

### Parameter configurations for the open-source LC/MS tools

XCMS, MZmine, and MAVEN were developed by three different groups and adopted different approaches. XCMS was developed in R and configured with two options, 'MatchedFilter' and 'centWave', to detect meaningful chromatographic peaks. MZmine was developed in Java with the 'centroidPicker' algorithm implemented for chromatographic peak detection. MAVEN was developed in C++ and equipped with a complex, machine learning-based peak filtering method. In addition, some of the configurable parameters for the three tools are slightly different. For example, XCMS and MZmine define peak width by scan units or minutes, respectively. Therefore, the software parameters must be carefully configured to produce comparable values. The scan rate for our analyzed UPLC/MS dataset is one scan per 1.2 seconds and we configured the optimal parameters for XCMS, MZmine, and MAVEN accordingly (Table 4).

**Table 4:** Parameter configurations for XCMS, MZmine, and MAVEN.

Methods	Parameters
XCMS	*Method: *"centWave" Mass resolution = 40 ppm, peakwidth = c(5,50)

MZmine	*Chromatograph parameters:* Noise level = 10, m/z tolerance = 0.08 or 40 ppm, Min time span = 0.1 min. *Peak deconvoluted parameters:* Min peak height: 50, Peak duration time range: 0.1-1.0 min.

MAVEN	*Feature detection parameters:* Mass resolution = 40 ppm, Time resolution = 3 scans. *EIC processing parameters:* EIC smoothing = 5 scans, Max group RtT difference = 0.1 min. *Peak scoring parameters* Peak classifier model=default model, Min. Good peak/group = 1, Min. signal/Base line ratio = 2, Min. peak width = 5 scans, Min. signal/Blank ratio = 2.00, Min. peak intensity = 100 ions.

### Ground truth peak definition

**Figure 6:**
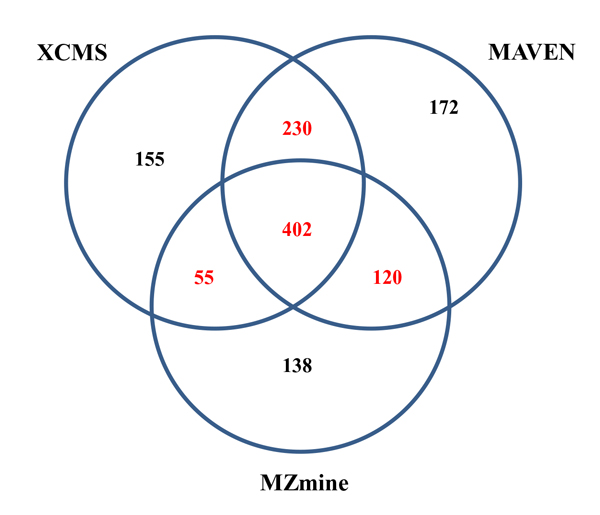
**Venn diagram of all detected peaks from the same UPLC data set by XCMS, MAVEN, and MZmine**. The overlapping (red colored) subsets were used as ground truth peaks.

We used the three tools to analyze the same UPLC/MS dataset and produced individual peak lists. Each peak is represented by its m/z and retention time value at the apex. Additionally, we specified 0.05 *Da *as the m/z tolerance and 5 seconds as the retention time tolerance to ensure that two peaks from two different software tools would be considered the same peak if they fall within the specified tolerance. Then, we analyzed the three output peak lists and counted the identical peaks detected by all three tools, those detected by two tools, and those detected by only one tool. Figure 6 shows a Venn diagram of the output peak analysis results from the three tools.

In this UPLC/MS dataset, 402 identical peaks were detected by all three tools, demonstrating their peak detection consistency. However, there were some identical peaks only detected by two of the three tools. Therefore a ground truth peak was defined as those peaks that were detected by at least two tools. We calculated the ground truth peak number using equation (9) and composed a ground truth peak list.

(9) $$\begin{array}{c} NP=N\left(MAVEN\cap MZmine\right)+N\left(MAVEN\cap XCMS\right)+N\left(MZmine\right)\cap XCMS)\\ -2N\left(MAVEN\cap MZmine\cap XCMS\right) \end{array}$$

Where, *N*(*MAVEN *∩ *MZmine *∩ *XCMS*) means the number of identical peaks detected by MAVEN and MZmine, and *N*(*MAVEN *∩ *MZmine *∩ *XCMS*) means the number of identical peaks detected by the all three tools. The ground truth peak list defined the authentic peaks contained in the dataset and were used to define the true positive peaks in our program.

If a peak detected by our program (represented as × in the following equations) was also found in the ground truth peak list, it was considered a true positive peak. The true positive peak number *TP *for our program was calculated using equation (10).

(10) TP(X)=N(X∩TruePeaklist)

Based on the ground truth peak definition and the true positive peaks, we can calculate the Recalls, Precisions and F-Scores for our program using equations (11)-(13).

(11) $$Recal\left(X\right)=\frac{TP\left(X\right)}{NP}$$

(12) $$Precision\left(X\right)=\frac{TP\left(X\right)}{NP}$$

(13) $$F-Score\left(X\right)=\frac{2Recall\left(X\right)*Precision\left(X\right)}{Recall\left(X\right)+Precision\left(X\right)}$$

We excluded our program from the ground truth peak definition, due to the varying metric cutoff thresholds for EIC and chromatographic peak quality evaluation. Using the three external tools with well-configured parameters provided stable peak detection results that could be used as a benchmark to assess our program and the quality metric's performance.

Additionally, using the Venn diagram (Figure 6), we calculated the peak detection performance for XCMS, MZmine and MAVEN, measured by Recalls, Precisions and F-Scores and listed in Table 5.

**Table 5:** Peak detection performance of XCMS, MZmine, and MAVEN.

Method	Recall	Precision	F-Score
XCMS	0.8513	0.7293	0.7856
MZmine	0.7150	0.8070	0.7582
MAVEN	0.9318	0.8139	0.8688

### Comprehensive evaluation and analysis of EIC quality metrics

**Figure 7:**
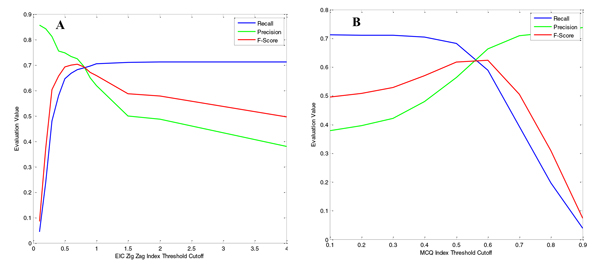
**EIC quality evaluation measured by the Recall, Precision, and F-Score**. The final detected peaks and the calculated Recall, Precision and F-Score are varying with the threshold cutoffs of the adopting metric of (A) the global zigzag index and (B) the MCQ index

Our program's final peak detection is affected by any of the four analysis modules: acute EIC extraction, EIC evaluation and filtering, chromatographic peak detection, and chromatographic peak evaluation and filtering, as seen in the LC-MS peak detection flowchart (Figure 1). In order to specifically investigate the relationship between EIC quality filtering and final peak detection performance, we fixed the parameters in other three modules and systematically varied the EIC quality evaluation filtering method and cutoff threshold. Then we calculated the corresponding Recalls, Precisions, and F-Scores to evaluate the final peak detection performance. The tests were performed using the existing MCQ index and our proposed zigzag index as the filtering method respectively. The final peak detection performance of the two EIC evaluation methods is shown in the Figure 7.

The results showed that as the cutoff threshold was loosened (increasing zigzag index threshold or decreasing MCQ index threshold), the Recall increased and Precision decreased accordingly; however, the F-Score, a more balanced evaluation value, increased fast initially and then decreased slowly. The maximum F-Scores for the two evaluation metrics appear at a zigzag index = 0.7 and MCQ index = 0.6, approximately. The maximum F-Score for the zigzag index evaluation method is obviously larger than for MCQ index method, which further validates the advantages of our proposed zigzag index for EIC quality evaluation.

### Comprehensive evaluation and analysis of chromatographic peak quality metrics

**Figure 8:**
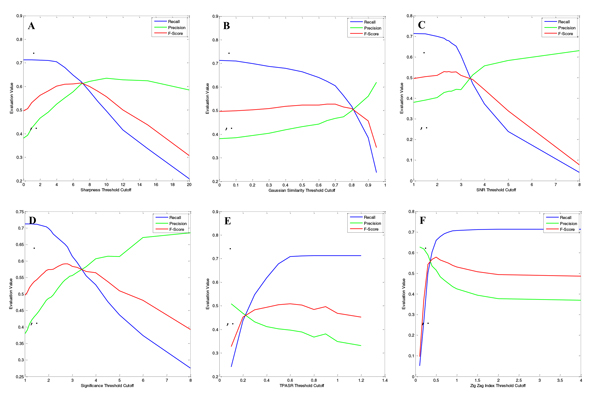
**Chromatographic peak quality evaluation measured by the Recall, Precision, and F-Score**. The final detected peaks and the calculated Recall, Precision and F-Score are varying with the threshold cutoffs of the adopting metric of (A) Sharpness, (B) Gaussian similarity, (C) SNR, (D) peak significance level, (E) TPASR, and (E) local zigzag index.

In the case-specific chromatographic peak evaluation, we have initially investigated six metrics for chromatographic peak quality evaluation. In order to specifically study the relationship between final peak detection performance and the individual peak quality metrics' cutoff thresholds, we bypassed the EIC quality filtering, selected one of the metrics, and adjusted its cutoff threshold systematically. We then calculated the corresponding Recalls, Precisions and F- Scores for the final peak detection performance analysis. The final peak detection performance plots are shown in Figure 8.

As seen with the EIC quality metrics, as the cutoff thresholds loosened, the Recall increased, the Precision decreased, and the F-Score increased to a maximum value, then decreased. Among the six chromatographic peak quality evaluation metrics, the F-Score curves for sharpness, peak significance level, and zigzag index have an obvious maxima and their F-Scores value are higher in magnitude than the other three metrics. The sharpness, peak significance level, and zigzag index metrics are advantageous for chromatographic peak quality evaluation because of the zigzag-like peak shapes observed in LC/MS chromatogram.

We also observed that all of the maximum F-Scores were smaller than 0.6. This is due to the bypassed EIC quality evaluation and use of a single metric to evaluate and filter the chromatographic peaks. However, if the six metrics are combined, it is very easy to achieve a relative high F-Score. For example, when we set Sharpness_Th = 2.0, Gaussian_Similarity_Th = 0.6, SNR_Th = 1.3, Peak_Significance_Th = 1.2, TPASR_Th = 0.8, Zigzag index = 0.9, we achieved a final Recall = 0.7076, Precision = 0.6186, and F-Score = 0.6601, which further supports our observation that several metrics should be used to evaluate chromatographic peak quality.

Additionally, when we selected the global zigzag index as the EIC evaluation metric in the EIC evaluation and filtering module and set the EIC_Zigzag index_Th = 0.9, we can achieved better peak detection performance with Recall = 0.6927, Precision = 0.7700, and F-Score = 0.7293.

## Conclusions

In this paper, we comprehensively investigated the quality evaluation metrics for the extracted EICs and the detected chromatographic peaks. For the EIC quality evaluation, we proposed a novel metric named the global zigzag index that can fairly evaluate the EICs with high background or containing only one single peak, in contrast to the existing MCQ Index. For the detected chromatographic peak quality evaluation, a comprehensive set of metrics including sharpness, Gaussian similarity, SNR, peak significance level, TPASR, and local zigzag index were analyzed and compared. Of the six peak quality metrics evaluated, the sharpness, peak significance level, and zigzag index outperformed the others due to the zigzag nature of LC/MS chromatographic peak shapes. Furthermore, we demonstrated that combining several peak quality metrics was more efficient than using an individual metric to evaluate the chromatographic peak quality.

Generally speaking, an ideal chromatographic peak should have a relatively high Gaussian- similarity, sharpness, SNR, peak significance, and a relatively low TPASR and zigzag index. While combining several metrics achieves better results, setting an optimal cutoff threshold for each metric still is a challenge task. Machine learning-based approaches, such as support vector machine (SVM), should be investigated to automatically identify good peaks in the future. This requires compiling and curating a sufficient number of "good" and "bad" representative peaks for training samples. From there the metrics described here could be used as input features for SVM model training. However, the presented quality evaluation metrics for extracted ion chromatograms and chromatographic peaks already demonstrate an significant improvement in quality peak detection and analysis. This represents a first step towards addressing the unique data analysis challenges seen with LC/MS-based metabolomics data.

## Availability and requirements

**Availability: **The LC/MS profile dataset and Matlab scripts are available upon request.

**Project name: **Quality evaluation of extracted ion chromatograms and chromatographic peaks in liquid chromatography/mass spectrometry-based metabolomics data.

**Operating system: **Platform independent.

**Programming language: **Matlab.

**Other requirements: **None.

**License: **None for usage.

**Any restrictions to use by non-academics: **None.

## Competing interests

The authors declare that they have no competing interests.

## Authors' contributions

PZ conceived and supervised the research and development of the project as well as the presented analyses. WZ developed the Matlab scripts and completed the metric performance evaluations. WZ and PZ wrote the manuscript. All authors read and approved the final manuscript.

## Declarations

Publication costs for this article were funded by the Samuel Roberts Noble Foundation.

This article has been published as part of *BMC Bioinformatics *Volume 15 Supplement 11, 2014: Proceedings of the 11th Annual MCBIOS Conference. The full contents of the supplement are available online at http://www.biomedcentral.com/bmcbioinformatics/supplements/15/S11.

## References

[B1] Murray Kermit K Boyd Robert K Eberlin Marcos N Langley GJ Li L Naito Y Definitions of terms relating to mass spectrometry (IUPAC Recommendations 2013) Pure and Applied Chemistry 2013 85 1515

[B2] Dettmer K Aronov PA Hammock BD Mass spectrometry-based metabolomics Mass spectrometry reviews 2007 26 1 51 78 10.1002/mas.20108 16921475 PMC1904337

[B3] Andreev VP Rejtar T Chen HS Moskovets EV Ivanov AR Karger BL A universal denoising and peak picking algorithm for LC-MS based on matched filtration in the chromatographic time domain Analytical chemistry 2003 75 22 6314 6326 10.1021/ac0301806 14616016

[B4] Lei Z Huhman DV Sumner LW Mass spectrometry strategies in metabolomics The Journal of biological chemistry 2011 286 29 25435 25442 10.1074/jbc.R111.238691 21632543 PMC3138266

[B5] Smith CA Want EJ O'Maille G Abagyan R Siuzdak G XCMS: processing mass spectrometry data for metabolite profiling using nonlinear peak alignment, matching, and identification Analytical chemistry 2006 78 3 779 787 10.1021/ac051437y 16448051

[B6] Tautenhahn R Bottcher C Neumann S Highly sensitive feature detection for high resolution LC/MS BMC bioinformatics 2008 9 504 10.1186/1471-2105-9-504 19040729 PMC2639432

[B7] Melamud E Vastag L Rabinowitz JD Metabolomic analysis and visualization engine for LC-MS data Analytical chemistry 2010 82 23 9818 9826 10.1021/ac1021166 21049934 PMC5748896

[B8] Windig W Phalp JM Payne AW A Noise and Background Reduction Method for Component Detection in Liquid Chromatography/Mass Spectrometry Analytical chemistry 1996 68 20 3602 3606 10.1021/ac960435y

[B9] Brodsky L Moussaieff A Shahaf N Aharoni A Rogachev I Evaluation of peak picking quality in LC-MS metabolomics data Analytical chemistry 2010 82 22 9177 9187 10.1021/ac101216e 20977194

[B10] Christin C Smilde AK Hoefsloot HC Suits F Bischoff R Horvatovich PL Optimized time alignment algorithm for LC-MS data: correlation optimized warping using component detection algorithm-selected mass chromatograms Analytical chemistry 2008 80 18 7012 7021 10.1021/ac800920h 18715018

[B11] Katajamaa M Miettinen J Oresic M MZmine: toolbox for processing and visualization of mass spectrometry based molecular profile data Bioinformatics (Oxford, England) 2006 22 5 634 636 10.1093/bioinformatics/btk039 16403790

[B12] Benton HP Wong DM Trauger SA Siuzdak G XCMS2: processing tandem mass spectrometry data for metabolite identification and structural characterization Analytical chemistry 2008 80 16 6382 6389 10.1021/ac800795f 18627180 PMC2728033

[B13] Stolt R Torgrip RJ Lindberg J Csenki L Kolmert J Schuppe-Koistinen I Jacobsson SP Second-order peak detection for multicomponent high-resolution LC/MS data Analytical chemistry 2006 78 4 975 983 10.1021/ac050980b 16478086

[B14] Christin C Hoefsloot HC Smilde AK Suits F Bischoff R Horvatovich PL Time alignment algorithms based on selected mass traces for complex LC-MS data Journal of proteome research 2010 9 3 1483 1495 10.1021/pr9010124 20070124

[B15] Choi D Row K Theoretical analysis of chromatographic peak asymmetry and sharpness by the moment method using two peptides Biotechnol Bioprocess Eng 2004 9 6 495 499 10.1007/BF02933492

[B16] Ni Y Qiu Y Jiang W Suttlemyre K Su M Zhang W Jia W Du X ADAP-GC 2.0: deconvolution of coeluting metabolites from GC/TOF-MS data for metabolomics studies Analytical chemistry 2012 84 15 6619 6629 10.1021/ac300898h 22747237

[B17] Kalambet Y Kozmin Y Mikhailova K Nagaev I Tikhonov P Reconstruction of chromatographic peaks using the exponentially modified Gaussian function Journal of Chemometrics 2011 25 7 352 356 10.1002/cem.1343

[B18] Du P Kibbe WA Lin SM Improved peak detection in mass spectrum by incorporating continuous wavelet transform-based pattern matching Bioinformatics (Oxford, England) 2006 22 17 2059 2065 10.1093/bioinformatics/btl355 16820428

[B19] Lange E Gropl C Reinert K Kohlbacher O Hildebrandt A High-accuracy peak picking of proteomics data using wavelet techniques Pacific Symposium on Biocomputing Pacific Symposium on Biocomputing 2006 243 254 17094243

[B20] Zhang W Chang J Lei Z Huhman D Sumner LW Zhao PX MET-COFEA: A Liquid Chromatography/Mass Spectrometry Data Processing Platform for Metabolite Compound Feature Extraction and Annotation Analytical chemistry 2014 86 13 6245 6253 10.1021/ac501162k 24856452

